# Different Models of Hospital–Community Health Centre Collaboration in Selected Cities in China: A Cross-Sectional Comparative Study

**DOI:** 10.5334/ijic.2456

**Published:** 2016-04-05

**Authors:** Jing Xu, Rui Pan, Raymond W. Pong, Yudong Miao, Dongfu Qian

**Affiliations:** School of Health Policy & Management, Nanjing Medical University, Nanjing, P.R. China; School of Public Health, Nanjing Medical University, Nanjing, P.R. China; Center for Rural and Northern Health Research, Laurentian University, Sudbury, Ontario, Canada

**Keywords:** integrated delivery systems, integrated care, health service system, chronic diseases, China

## Abstract

**Objective:**

In recent years, in order to provide patients with seamless and integrated healthcare services, some models of collaboration between public hospitals and community health centres have been piloted in some cities in China. The main goals of this study were to assess the nature and characteristics of these collaboration models.

**Methods:**

Three cases of three different collaboration models in three Chinese cities were selected to analyse using descriptive statistics, Pearson *χ*^2^ and ordinal logistic regression.

**Results:**

Results showed that the Direct Management Model in Wuhan exhibited better structure indicators than the other two models. Staff in the Direct Management Model had the highest satisfaction level (77.6%) with respect to patient referral. Communications between hospitals and community health centres and among care providers were generally inadequate. Publicity about hospital–community health centre collaboration was inadequate, resulting in low awareness among patients and even among health professionals.

**Conclusion:**

Results can inform health service delivery integration efforts in China and provide crucial information for the assessment of similar collaborations in other countries.

## Introduction

To overcome the problem of fragmentation in health service system, integrated health services delivery has been proposed to provide patients with a seamless and interconnected healthcare model, which has become a new trend in international healthcare reforms. Close cooperation between hospitals and community health centres is seen as a suitable way to deliver chronic disease services [1]. The disease spectrum of populations in developed and many developing nations, including China, has changed substantially. Chronic diseases are now the major cause of disability, principal reason for visiting physicians and cause for 70% of healthcare expenditures [2]. Nowadays, health service integration, especially concerning chronic diseases, is one of the major tasks of health system reform in many countries [3] and is also seen as a way to improve quality of care and lower healthcare costs [4,5].

In urban areas in China, health services are provided by healthcare institutions at different levels, including community health centres, secondary hospitals and tertiary hospitals. Most community health centres are established by the government, though a few are created by the private sector. As the main provider of primary health care in urban areas, they provide services in six areas: preventive services, medical care (including inpatient care), health services, health education, rehabilitation services and family planning guidance. Most secondary and tertiary hospitals in China are public. However, most community health centres have no regular collaboration relationship with hospitals. Generally speaking, these community health centres do not receive technology or management support (such as green channel of reciprocal referrals, specialist outreach service, medical service training and guidance) from hospitals. There is also a lack of effective communication and cooperation among institutions at different levels and there may even be competitions between them [6]. As a result, it is difficult to have coordinated services and continuity of care across different care settings. To overcome this problem, initiatives have been introduced to build a more integrated healthcare delivery system, which has become one of the most important objectives of the current Chinese healthcare reform.

With a view to integrating urban healthcare resources, China began, in 1997, to construct a two-level healthcare system consisting of tertiary hospitals (large general hospitals) and community health centres. Since the promulgation of the ‘Guidance on the Development of Urban Community Health Services’ by the State Council (or Cabinet) of China in 2006, many secondary hospitals have been transformed into community health centres. By the end of 2008, community health centres could be found in all cities and 98% of municipal districts nationwide [7]. As the government has produced some policies to promote the collaboration between hospitals and community health centres, some collaboration models between hospitals and community health centres have been unfolded in some cities. At present, the models of collaboration between hospitals and community health centres in China mainly include the following three kinds of type: Loose Collaboration Model, Medical Consortium Model and Direct Management Model. The above models are approximately in correspondence with liaison model, coordination model and full-integration model, respectively, that were suggested by Leutz [8]. The participants in collaboration models mainly involve public tertiary general hospitals and community health centres, which are the main interagency collaborations between hospitals and community health centres in urban China [8].

Loose Collaboration Model is the most common in China nowadays. In this model, through an agreement, loose and flexible cooperative relationship between a hospital and related community health centres is established. Hospital does not get involved in the management of community health centres and just provides some medical service collaboration or support for community health centres. The next most common is the Medical Consortium Model, which typically involves one tertiary hospital as the core institution and other healthcare agencies, such as community health centres, as partners. These organisations are integrated into a ‘medical consortium’ through some mechanisms such as funding allocations or formal contracts. The exact number of medical consortiums is unknown, but we estimate there are dozens of them in China [9]. In the Medical Consortium Model, the medical group owns the right of management over related community health centres, except the personnel appointment. Direct Management Model is the least common form of hospital–community health centre collaboration and there are only a few in the whole country [10,11]. In the Direct Management Model, the hospital is entrusted by the local government the responsibility of administration and operations concerning personnel, finance, health resources and management of affiliated community health centres. Hospital owns the rights of personnel appointment and management control over community health centres. The hospital could appoint and supervise community health centre personnel. In a way, the community health centres could be seen as departments of the hospital [12]. Specific characteristics of these three collaboration models will be discussed in greater detail in the third section where three selected collaborations are described.

While some hospital–community health centre collaborations exist in China, there has been little systematic effort to evaluate them. There are a few mostly descriptive studies. For example, Liu et al. [13] described the collaboration between Peking University People’s Hospital and two community health centres, suggesting that it was a ‘loose collaboration’ that could save patients’ visit time and drug costs. Tang et al. [14] studied the collaboration between hospitals and community health centres in Shanghai and Beijing, respectively. However, they have largely failed to provide strong empirical evidence regarding the nature and characteristics of such collaborations that is the main goals of this study.

Generally speaking, little work has been done to examine and analyse the status of different models of hospital–community health centre collaboration in China. Since vertical integration of health service delivery is a key strategy in reforming health systems around the world [15], it is imperative to find out how hospital–community health centre collaborations work and how they perform in China.

## Methodology

### Design

The structure-process-outcome framework by Donabedian [16] provides the conceptual framework for this study. Structure refers to the underpinning infrastructure and resources that an organization has in place to achieve its aims (personnel, resources, policies and operational procedures). Process refers to what it actually does and outcome refers to the results of what it does [17,18]. On the basis of this framework, related ‘structure, process, and outcomes’ of three different collaboration models were investigated and analysed in this study.

Related collaboration models have been conducted in China before this study and corresponding intervention researches were very difficult to implement in fact. Therefore, a cross-sectional survey of three hospital–community health centre collaborations in three cities was designed. Some indicators of ‘structure, process, and outcomes’ of three different collaboration models were also difficult to investigate. Thus, related descriptive statistics and Pearson *χ*^2^ inspection were mainly used for comparative analysis of three models. Because patient satisfaction was measured in terms of ‘satisfied’, ‘neither satisfied nor dissatisfied’ and ‘dissatisfied’, we still chose ordinal logistic regression to further analyse patients’ satisfaction with their treatment.

### Samples and data collection

Using a natural experiment approach, this study compared three typical cases – one sample for each of three models – in three Chinese cities. Stakeholder analysis was conducted in each model. In this study, stakeholders involved all health professionals who participated in collaboration work and patients from sample community health centres. In our study, all sample community health centres and tertiary general hospitals are established and financed by the government.

Direct Management Model has been piloted in Wuhan, Hubei Province since 2009 and up to when this survey there is no other strict similar case in China. The Medical Consortium Model of collaboration between a tertiary hospital and several community health centres in Zhenjiang, Jiangsu Province was generally believed that is the best representative for Medical Consortium models in China at present. Therefore, the Direct Management Model in Wuhan and the Medical Consortium Model in Zhenjiang were selected as study subjects in this research. According to the comparability and simple random sample, we selected Loose Collaboration Model of collaboration between a tertiary hospital and several community health centres in Nanjing, Jiangsu Province as the control group to analyse.

Each of the selected collaboration involved one hospital and multiple community health centres. Therefore, in addition to surveying the selected hospital, we used a stratified random sampling method to select three community health centres within each collaboration model and patients within the sampled community health centres for inclusion in the survey. Within each model, all health professionals who participated in health service delivery collaboration were selected for the survey. As patients with Type 2 diabetes and hypertension often seek treatment at both hospitals and community health centres, these two chronic diseases were regarded as appropriate tracer diseases for this study. Thus, outpatients with Type 2 diabetes and hypertension who were visiting doctors at the sampled community health centres during the survey period were selected.

The inclusion criteria for patients with Type 2 diabetes or hypertension were the following: (1) had received medical service from both community health centre physicians and hospital specialists during a 1-year period preceding the day of the survey, (2) were aged 18–80; (3) were permanent residents of the study communities and (4) consented to participate in the study. But some patients were excluded. The exclusion criteria were the following: (1) Those with severe diabetic or hypertension complications (e.g. diabetic foot or severe diabetic retinopathy or severe hypertension complications) and (2) those with terminal illness (e.g. with terminal cancer or AIDS).

A review of institution documents and reports was conducted to collect information about the operations of the sampled hospitals and community health centres. A questionnaire survey was also conducted at each of the sampled institutions. The questionnaire for tertiary hospital and community health centres covered basic conditions of the institutions, nature of the collaborations, etc. The survey was conducted by Nanjing Medical University during September 2012.

To acquire the perception of stakeholders, survey for patients and health professionals was by means of a semistructured questionnaire designed to fulfil the study aims. The interview questionnaire for patients covered medical procedures, perceived quality of care, satisfaction and attitudes in relation to outpatient care and the interface between hospitals and community health centres. The interview questionnaire for hospital and community health centre health professionals covered a range of issues including collaboration models, relationships between community health centre physicians and hospital specialists, and the process and outcomes of the collaboration.

After obtaining informed consent from the research subjects, we distributed 174 questionnaires to health professionals and received 174 completed questionnaires, achieving a 100% response rate. We surveyed 1365 community health centre patients and 1254 questionnaires were fully completed, achieving a response rate of 91.9%.

### Research ethics approval

The participant consents were documented in the first section of each structured questionnaire and all illiterate respondents gave verbal informed consent before participating in this study. No study interventions were performed and all data collected were anonymous statistical data. Participants were assured that the information they gave would be used solely for the purposes of this study and they did not display apprehension in this regard. The Research Ethics Committee of Nanjing Medical University approved the study and its consent procedure.

## Results and analysis

### Characteristics of sampled patients

The characteristics of the sampled patients were shown in Table 1. The marital status variable was divided into two categories: married and others (including single, divorced, separated and widowed). Severity of illness, which was self-defined by the respondents, was classified as low, medium and high.

**Table 1:** Characteristics of sampled patients in three collaboration models.

Characteristic	Direct Management (Wuhan)% (*N*)	Medical Consortium (Zhenjiang)% (*N*)	Loose Collaboration (Nanjing)% (*N*)	Total% (*N*)	*χ*^2^ test

Sex					
Male	42.3% (160)	44.1% (158)	49.6% (176)	45.3% (494)	*χ*^2^ = 4.165
Female*	57.7% (218)	55.9% (200)	50.4% (179)	54.7% (597)	*P* = 0.125
Age					
18–60*	15.1% (57)	29.9% (107)	33.8% (120)	26.0% (284)	*χ*^2^ = 40.923
61–70	44.2% (167)	41.3% (148)	34.1% (121)	40.0% (436)	*P* = 0.000
71–80	40.7% (154)	28.8% (103)	32.1% (114)	34% (371)	
Marital status					
Married	91.3% (345)	93.3% (334)	93.5% (332)	92.7% (1011)	*χ*^2^ = 1.676
Others*	8.7% (33)	6.7% (24)	6.5% (23)	7.3% (80)	*P* = 0.433
Educational level					
Illiterate/elementary*	31.0% (117)	50.8% (182)	15.5% (55)	32.4% (354)	*χ*^2^ = 137.963
Middle/high school	52.9% (200)	40.5% (145)	50.4% (179)	48.0% (524)	*P* = 0.000
College or above	16.1% (61)	8.7% (31)	34.1% (121)	19.5% (213)	
Severity of illness					
Low	54.2% (205)	46.6% (167)	39.2% (139)	46.8% (511)	*χ*^2^ = 20.926
Medium	42.3% (160)	46.1% (165)	52.4% (186)	46.8% (511)	*P* = 0.000
High*	3.4% (13)	7.3% (26)	8.5% (30)	6.3% (69)	
Types of disease					
Type 2 diabetes mellitus	19.8% (75)	24.6% (88)	31.5% (112)	25.2% (275)	*χ*^2^ = 13.421
Hypertension*	80.2% (303)	75.4% (270)	68.5% (243)	74.8% (816)	*P* = 0.001

Note: Figures in brackets refer to corresponding frequency. *Indicates the omitted group in the *ordinal logistic* regression.

### Characteristics of health professionals

There were 58 health professionals in the Direct Management Model, 63 in the Medical Consortium Model and 53 in the Loose Collaboration Model, accounting for 33.3%, 36.2% and 30.5%, respectively. The education degree was divided into three categories: College (no bachelor’s degree), bachelor and postgraduate. Professional title was classified as senior, middle-level and junior or below. The characteristics of the health professionals were shown in Table 2. The results showed that there was no statistically significant difference between the categories of every characteristic at the 0.05 level.

**Table 2:** Characteristics of sampled health professionals in three collaboration models.

Characteristic	Direct Management (Wuhan)% (*N*)	Medical Consortium (Zhenjiang)% (*N*)	Loose Collaboration (Nanjing)% (*N*)	Total % (*N*)	*χ*^2^ test

Sex					
Male	34.5% (20)	20.6% (13)	22.6% (12)	26.5% (45)	*χ*^2^ = 3.433
Female	65.5% (38)	79.4% (50)	77.4% (41)	73.5% (129)	*P* = 0.180
Age					
20~30	39.7% (23)	52.4% (33)	30.2% (16)	43.6% (72)	*χ*^2^ = 7.113
31~40	36.2% (21)	34.9% (22)	45.3% (24)	35.2% (67)	*P* = 0.130
41 or over	24.1% (14)	12.7% (8)	24.6% (13)	21.2% (35)	
Education degree					
College	30.0% (18)	47.6% (30)	39.6% (21)	40.1% (69)	*χ*^2^ = 4.927
Bachelor	56.9% (33)	42.9% (27)	43.4% (23)	47.1% (83)	*P* = 0.29
Postgraduate	12.1% (7)	9.5% (6)	17.0% (9)	12.8% (22)	
Occupation					
Physician	48.3% (28)	39.7% (25)	50.9% (27)	46.0% (80)	*χ*^2^ = 1.655
Nurse	51.7% (30)	60.3% (38)	49.1% (26)	54.0% (94)	*P* = 0.437
Professional title					
Senior	43.1% (25)	57.1% (36)	43.4% (23)	48.3% (84)	*χ*^2^ = 7.946
Middle level	51.7% (30)	42.9% (27)	47.2% (25)	47.1% (68)	*P* = 0.080
Junior or below	5.2% (3)	0 (0)	9.4% (5)	4.6% (8)	

### General characteristics of three collaboration models

There was a weak binding force in Loose Collaboration Model because of the loose collaboration. Major forms of collaboration involve medical specialists from hospitals providing outpatient services at community health centres, reciprocal referrals and so on. Usually, their collaboration follows the guidance of health administrative departments and depends on the needs of community health centres. However, because culture integration is weak, such collaborations usually lack structured and effectual management support. The work of collaboration mainly depends on the familiarity and relationships of related personnel. What is more, in this model, each of related institutions is still independent and is responsible for their own development, which means competition still exists among them.

In the Medical Consortium Model, hospital would generally provide related community health centres with some devices supported. Related community health centres would be affected by the brand of medical group. Equipment adjustment could be carried out within group, and communication within group is convenient. Within the group, an established department is responsible for reciprocal referrals. However, while the medical group takes charge of the development of community health centres, the hospital mainly concentrates on the development of itself.

In the Direct Management Model, the leader of the hospital is completely responsible for the development of community health centres and its interests. Besides, related community health centres could recruit workers in the name of the hospital. Therefore, hospital’s concepts can infuse in community health centres conveniently and resource allocation among them is convenient. Generally, community health centres could be seen as a department of a hospital.

The general characteristics of the three collaboration models are highlighted in Table 3. The three models of hospital–community health centre collaboration shared some common characteristics. For example, there was no change in the legal status of the community health centres between pre- and post-collaboration implementation. There was also no change in ownership of community health centres and oversight roles over community health centre in three models, which is the same and is owned by the local government. In addition, medical specialist outreach services, defined as planned, regular visits by hospital specialists to see patients in community health centres [19], occurred in each model.

**Table 3:** Comparisons of structure of three collaboration models.

Dimensions/indicators	Direct Management Model (Wuhan)	Medical Consortium Model (Zhenjiang)	Loose Collaboration Model (Nanjing)

Ownership of community health centre		Same (the local government)	
Oversight roles over community health centre		Same (the local government)	
Operation responsibilities over community health centre	Hospital	Community health centre	Community health centre
Financing source of community health centre	Hospital, Local government, Medical service and other revenue	Local government, Medical service and other revenue	Local government, Medical service and other revenue
Personnel recruitment and management over community health centre	Hospital	Community health centre	Community health centre
Collaboration implementation cost	+++	+++	+
Degree of shared financial resources	++	++	+
Degree of healthcare resources integration	+++	++	+
Extent of interagency integration	+++	++	+
Degree of patient information sharing between hospital and community health centres	++	++	+
Degree of sharing of organisational culture	+++	++	+
Adherence to public health service goal	+	++	+++
Organisational structure: Administrative authority over community health centres	Hospital	Hospital	community health centres
Financing			
Average annual subsidy for hospital from local government from 2009 to 2011 (Yuan)	1.1 Million	0	0
Facility/equipment:			
1 Average number of beds at each community health centres	79.5	28.7	79.3
2 Average number of beds at hospital	800	1030	1000
3 Establishing an information-sharing platform between hospital and community health centres	Yes	Yes	Yes
Staffing:			
Special office and staff to oversee collaboration and management	Yes	Yes	No
Policy			
1 Incentives to promote institutional collaboration	Yes	Yes	Yes
2 Whether to have health insurance incentives	No	Yes	No

Note: ‘+‘means least or weakest; ‘+++’ means most or strongest.

There were also some differences between the three collaboration models. In the Direct Management Model in Wuhan, the core hospital had direct jurisdictional control over the affiliated community health centres, including operation responsibilities, personnel recruitment and management, and material resources utilisation over community health centres, which are both different with the other two models. Financing source of community health centres in the Direct Management Model include hospital (about 5.0%), local government (about 34.0%), medical service revenue and other revenue (about 61.0%), which, however, include only local government (about 34.0%), medical service revenue and other revenue (about 66.0%) in the other two models.

In the Medical Consortium Model in Zhenjiang, rather than direct management, the core hospital provided the affiliated community health centres with operational guidance and shared some equipment and other resources. With respect to administration, the hospital provided management support but could not appoint or manage community health centre personnel.

In the Loose Collaboration Model in Nanjing, a mutually agreed-upon relationship was established through an agreement between the hospital and the community health centres, but the former was not involved in the management of the latter. The hospital simply provided some medical services and other kinds of support to the community health centres.

### Structure analysis – three collaboration models

Evaluation of the structure included five aspects: organisational structure, financing, facility/equipment, staffing and policy. For different institutions to work effectively in collaboration, proper infrastructure and financing support were important. In 3 years from 2009 to 2011, the only hospital that received financial support from the local government was the one in the Direct Management Model, and the average amount was about RMB 1.13 million ($ 0.18 million). The average number of inpatient beds in a community health centre was about 80 in the Direct Management Model and Loose Collaboration Model, while it was about 29 in the MC Model. Moreover, there were some incentive policies for collaboration in each model. For example, community health centres could recruit staff in the name of hospital in the Direct Management Model. To encourage patients with chronic diseases to be willing to receive treatment in community health centres, Medical Consortium Model had an incentive policy in terms of health insurance: chronic patients insured by China basic medical insurance system for urban employees will be reimbursed for 90% of their medical expense by health insurance when receiving treatment in community health centres if their medical expense exceeds payment capability of individual account.

One of the main functions of community health centres in China is to provide public health services, including health promotion, disease prevention, immunization, prevention of infectious diseases, maternal and child health, etc. Under the Loose Collaboration Model, community health centres operated independently, but local district government supervises the operation and provision of public health services. Under the Medical Consortium Model, hospital can provide more guidance about public health service delivery for community health centres but local district government has the same supervision responsibility. Under the Direct Management Model, the hospital may use the allied community health centres as a marketing strategy to expand its medical service market because the community health centres are under minimal supervision from government.

### Process and outcome analysis

Our evaluation of process and outcomes of the collaborations focuses on the following aspects: (1) specialists involvement, such as number, diversity, frequency of attendance and turnover; (2) information exchanges and their contents; (3) number and waiting time of reciprocal referrals; (4) doctors’ and patients’ views on collaboration.

According to institution documents and reports of the hospitals and community health centres, in 2011, an average of 3.7 hospital specialists outreached to each community health centre and each outreach specialist spent an average of 1.88 days per week at the community health centres in the Medical Consortium Model, which was higher than in the Loose Collaboration Model, but lower than in the Direct Management Model.

Between 2008 and 2011, the percentage increases in patient referrals to hospitals from community health centres and to community health centres from hospitals in the Direct Management Model were higher than the Loose Collaboration Model.

The survey of health professionals showed that, in terms of their awareness of the nature of the collaboration, the differences of proportions among the three collaboration models were statistically different (*χ*^2^ = 19.777, *P* = 0.000). Pairwise comparisons between groups using SNK-q test showed that the Direct Management Model was different from the Medical Consortium Model (*χ*^2^ = 15.626, *P* = 0.000) and the Loose Collaboration Model (*χ*^2^ = 17.756, *P* = 0.000), but there was no statistically significant difference between the Medical Consortium Model and the Loose Collaboration Model (*χ*^2^ = 0.164, *P* = 0.686).

The knowledge level of patients regarding the nature of hospital–community health centre collaboration in the Direct Management Model and Medical Consortium Model was lower than that in the Loose Collaboration Model. With respect to how patients viewed their physicians’ understanding of their health problems, the proportion of patients who thought that the community health centre doctors knew their medical history was 45.7% in the Direct Management Model, which was the lowest among the three models. On the contrary, the proportion of patients who thought that hospital specialists knew their medical history was 31.4% in the Direct Management Model, which was the highest among the three models; however, the results in Table 4 showed no significance in this indicator.

**Table 4:** Indicators of the process of collaboration in the three models, 2011.

Dimension	Indicator	Direct Management % (*N*)	Medical Consortium % (*N*)	Loose Collaboration % (*N*)	*χ*^2^ test

Medical care	Average number of hospital departments participating in collaboration with community health centres	21	7	8	–
	Average number of outreach specialists from hospital providing medical care to patients at community health centres	4	3.7	3.3	–
	Average amount of time (days) spent by each outreach specialist at community health centres per week	1.9	1.8	1	–
Workload	Percentage increase in community health centre outpatients between 2008 and 2011 (%)	45.2%	79.9%	40.3%	–
Training of health workers	Average number of health professionals per year from community health centres receiving training in hospital in 3 years from 2009 to 2011	8	3.7	2.7	–
Referral	Percentage increase in patient referrals to hospitals from community health centres between 2008 and 2011	86.7%	–*	26.9%	–
	Percentage increase in patient referrals to community health centres from hospitals between 2008 and 2011	133.3%	–*	47.4%	–
Stakeholders’ perceptions	Percentage of health professionals who were aware of the nature of the collaboration	91.4% (53)	60.3% (38)	56.6% (30)	*χ*^2^ = 19.777 *P* = 0.000
	Percentage of patients who knew about the nature of hospital–community health centre collaboration.	20.8% (71)	24.5% (61)	41.4% (144)	*χ*^2^ = 39.014 *P* = 0.000
	Percentage of patients who thought community health centre doctors knew their medical history	45.7% (156)	69.5% (173)	61.8% (215)	*χ*^2^ = 36.522 *P* = 0.000
	Percentage of patients who thought specialists from hospitals knew their medical history	31.4% (107)	27.7% (69)	25.6% (89)	*χ*^2^ = 2.911 *P* = 0.233

Note: Figures in brackets are corresponding frequencies. *Data are not available due to lack of record.

Patients’ perceptions of their health conditions partly reflected their satisfaction with the quality of service they have received [20]. Our results showed that there were statistically significant differences among the three models (*χ*^2^ = 31.614, *P* = 0.000).

Satisfaction with hospital–community health centre collaboration was examined from the perspectives of patients and health professionals (see Table 5). A total of 651 patients responded that they did not know whether the hospital doctors and community health centre doctors had communicated with each other about their illness or health conditions. The *χ*^2^ test showed that there were no significant differences (*χ*^2^ = 1.081, *P* = 0.583) among the three models. Similarly, there were no differences in relation to patients’ satisfaction with treatment effects (*χ*^2^ = 4.111, *P* = 0.391).

**Table 5:** Patients’ and health professionals’ assessments on health and other outcomes.

Patients’ perception on communications between hospitals and related community health centres regarding patients’ illness			

	Sufficient % (*N*)		Not sufficient % (*N*)	

Direct Management Model	18.9 (21)		81.1 (90)	*χ*^2^ = 1.081
Medical Consortium Model	16.4 (27)		83.6 (138)	*P* = 0.583
Loose Collaboration Model	14.3 (25)		85.7 (150)	
Sub-Total	16.2 (73)		83.8 (378)	
**Patients’ satisfaction with treatment**				
	**Satisfied % (*N*)**	**Medium % (*N*)**	**Dissatisfied % (*N*)**	***χ*^2^ test**
Direct Management Model	57.4 (220)	37.1 (142)	5.5 (21)	*χ*^2^ = 4.111
Medical Consortium Model	64.1 (236)	31.3 (115)	4.6 (17)	*P* = 0.391
Loose Collaboration Model	61.6 (229)	32.5 (121)	5.9 (22)	
Sub-Total	61.0 (685)	33.7 (378)	5.3 (60)	
Health professionals’ satisfaction with collaborative treatment of patients,% (*N*)				
Direct Management Model	69.0 (40)	25.9 (15)	5.1 (3)	*χ* ^2^ = 8.094
Medical Consortium Model	44.5 (28)	44.4 (28)	11.1 (7)	*P* = 0.083*
Loose Collaboration Model	50.0 (26)	42.3 (22)	7.7 (4)	
Sub-Total	54.3 (94)	37.6 (65)	8.1 (14)	
Health professionals’ satisfaction with referrals of patients,% (*N*)				
Direct Management Model	77.6 (45)	22.4 (13)	0.0 (0)	*χ* ^2^ = 16.625
Medical Consortium Model	54.0 (34)	36.5 (23)	9.5 (6)	*P* = 0.001*
Loose Collaboration Model	48.1 (25)	50.0 (26)	1.9 (1)	
Sub-Total	60.1 (104)	35.8 (62)	4.1 (7)	
Health professionals’ satisfaction with communication about patients’ illness,% (*N*)				
Direct Management Model	62.1 (36)	34.5 (20)	3.4 (2)	*χ* ^2^ = 7.908
Medical Consortium Model	41.3 (26)	44.4 (28)	14.3 (9)	*P* = 0.91*
Loose Collaboration Model	44.2 (23)	44.2 (23)	11.6 (6)	
Sub-Total	49.2 (85)	41.0 (71)	9.8 (17)	

*Results of Fisher’s exact test.

However, there were statistically significant differences among the three models in relation to health professionals’ satisfaction with respect to referrals of patients. In the Direct Management Model, 77.6% of the health professionals were satisfied with patient referrals, which was considerably higher than the satisfaction in the other two models. Pairwise comparisons between models show that there were significant differences between the Direct Management Model and the Medical Consortium Model (*P* = 0.004) and between the Direct Management Model and the Loose Collaboration Model (*P* = 0.002). However, there was no significant difference between the Medical Consortium Model and the Loose Collaboration Model (*P* = 0.139) (see Table 5).

The results of the parameter estimates of ordinal logistic regression (see Table 6) showed that sex, education, age, marital status and type of disease did not affect satisfaction. However, the relationships between satisfaction and severity of disease and types of model were statistically significant. More specifically, patients whose disease was of ‘low severity’ were 29.6% more likely to be ‘satisfied’ than those with ‘high severity’. And patients whose disease was of ‘medium severity’ were 67.2% were more likely to be more satisfied than those with ‘high severity’. Patients from the Direct Management Model had odds of being in the ‘satisfied’ category that was 28.9% less than those from Loose Collaboration Model.

**Table 6:** Parameter estimates (*ordinal logistic* regression).

			95% CI	
			
Variables	*B* (SE)	OR	Lower bound	Upper bound

Sex				
Male	–0.159 (0.130)	0.853	0.662	1.100
Age				
61–70	0.211 (0.167)	1.235	0.890	1.714
71–80	0.205 (0.158)	1.228	0.900	1.673
Marital status				
Married	–0.110 (0.237)	0.896	0.563	1.424
Education level				
Middle/high school	0.105 (0.153)	1.111	0.824	1.499
College and above	0.177 (0.196)	1.194	0.812	1.755
Severity of illness				
Low	1.667*** (0.253)	5.296	3.224	8.707
Medium	0.983*** (0.247)	2.672	1.649	4.334
Types of disease				
Type 2 diabetes mellitus	–0.073 (0.143)	0.930	0.703	1.230
Model^a^				
Medical Consortium Model	0.129 (0.164)	1.138	0.825	1.570
Direct Management Model	–0.341** (0.157)	0.711	0.522	0.967

Note: ^a^Loose Collaboration Model was taken as a controlled group. **Indicates significance at 5%. ***Indicates significance at 1%.

## Discussion

Chronic noncommunicable diseases have such characteristics as long duration, multiple causation, undulating course and hard to cure [1]. Therefore, in dealing with chronic diseases, healthcare organisations have paid special attention to the way treatments and care are provided, and integration of health services delivery has received increased attention [4]. Dove et al. [21] have pointed out that integrated healthcare delivery is currently the favoured approach to make the best use of health resources and to reduce cost. Diana et al. [22] compared the process of two very different collaboration models in Boston, to gain a deeper understanding of a hospital’s experience living through such a change. Oelke et al. [23] have described the planning and implementation of a hospital–community collaboration model in Alberta, Canada, and how the model has improved quality of patient care and efficiency of service delivery. In China, strengthening the collaboration between hospitals and community health centres is thought to be critical in China’s current healthcare reform. Collaboration models involving hospitals and community health centres have been launched in some cities to ensure better coordination, efficiency and quality. Our research was to describe the characteristics of three different models of hospital–community health centre collaboration and to examine and compare some differences in the implementation and effects of health care for patients with chronic disease.

To avoid potential confounding factors caused by different diseases, we focused on patients with Type 2 diabetes mellitus and hypertension. Also, to avoid outlier effects, we excluded patients with serious complications and comorbidities. In addition, we elaborated the meaning and the purpose of the survey to all possible respondents before the face-to-face survey, and therefore, the response rates were high (health professionals 100.0% and patients 91.9%). We noticed that the Direct Management institution cared for a much older population because of the fact that the Direct Management Model was in Hanyang district, an old industrial region with more residents aged 60 or over of Wuhan. We tested its impacts on knowing about the nature of hospital–community health centre collaboration, thinking that community health centre doctors know their medical history and thinking that specialists from hospitals know their medical history, but no statistical difference was found (*P* > 0.05). In addition, we found that education degree was related to the knowledge about the nature of hospital–community health centre collaboration. The percentage of Loose Collaboration Model respondents who knew about the nature of hospital–community health centre collaboration was higher than the Direct Management and Medical Consortium Models (*χ*^2^ = 24.125, *P* = 0.000), which suggested that the publicity should emphasise on patients with less education.

Our study showed that, on the whole, the Direct Management Model in Wuhan had better ‘structure’ indicators than the other two models, which is likely attributed to its closer relationship in collaboration. In the Direct Management Model in Wuhan, the hospital is responsible for the operation responsibilities, personnel recruitment and management, and material resources utilisation over community health centres, which can contribute to improving resource allocation efficiency and strengthening the cooperation between hospital and community health centres. On the other hand, Direct Management Model has also its own weaknesses such as strong dependency on support degree of hospital leaders, possible inconsistency on public health service goal or task between hospital and community health centres, and so on. In terms of ‘process’ and ‘outcomes’, the survey of stakeholders’ perception showed that there were no significant differences in patients’ perceptions of communications between hospitals and community health centres regarding their illness, although health professionals’ satisfaction with collaborative treatment and communication regarding patients’ illness was higher in the Direct Management Model than in the other two models. Most patients thought the communication was not sufficient. Many reasons may result in it. For example, the low level of information technology cannot provide different institutions with tools to be in contact with each other effectively [24]. And due to inadequate publicity about hospital–community health centre collaboration, the awareness about the collaboration model was low among patients and even health professionals.

Patients’ satisfaction from the Direct Management Model was less than those from Loose Collaboration Model, which may be caused by its own weaknesses in the former but it needs further investigations in the future. In general, patients’ satisfaction with the effect of treatment was not high in all models, suggesting that all three models need to take measures to improve their collaborative services. Two factors, including severity of disease and types of model, affected their satisfaction.

Perfecting the promotion mechanism is the most important thing that should be emphasized, especially the benefit-sharing mechanism among different stakeholders, which could align the interests of hospital with community health centres and patients and make them become a community of interest. It can help arouse each stakeholder’s enthusiasm to work for cost-effective and cost–benefit health service. Meanwhile, cultural integration across institutions with different levels should be strengthened. For example, it is necessary to establish mutual support and trust among doctors from different institutions, which would help carry out the collaboration in practice rather than be set on one side.

This study has some limitations, most of which are a function of the nature of the research. Although related descriptive statistics and quantitative methods were used in this study, the analysis on three models is still not thorough and comprehensive. The data in this study came from a cross-sectional survey of three hospital–community health centre collaborations in three cities in China, which limits the use or obtains of other research methods and results. Although the Direct Management Model appears to have outperformed the other two, we cannot say for sure whether this is due to the superiority of the Direct Management Model or to this particular hospital–community health centre collaboration or to its location (Wuhan) or to a combination of these factors. Future studies should involve a much larger number of collaborations selected randomly from across the country.

## Recommendations

### Further research on different collaboration models

The current study should be seen as exploratory in nature. Nevertheless, it offers some interesting insights about hospital–community health centre collaborations, upon which hypotheses could be formulated for future testing. Because hospital–community health centre collaborations are not extensively studied in China and because our findings suggest that different models of collaborations may yield different performance outcomes, further research to examine the strengths and weaknesses of different models could help guide the future development of hospital–community health centre collaborations, which appear to be a viable approach for providing care to patients with chronic conditions.

### Strengthen information sharing and communication

Productive collaboration between different healthcare agencies or involving agencies at different levels requires effective communication and sharing of information. However, communications between hospitals and community health centres and among care providers were generally inadequate. Improving communication and information flow within a collaboration, including sharing of information about patients when they are referred from hospital to community health centre or vice versa is one of the top priority issues. But this is not just a task for individual health-care providers; it requires institutional attention and support.

### Pay attention to health professionals’ awareness

Doctors are one of the key stakeholders in hospital–community health centres collaboration. However, our study has shown that many of them were not knowledgeable about the nature of the collaboration and their enthusiasm was not high. To ensure the future success of collaborations, it is important to ensure that physicians, as well as other healthcare providers, know more about the collaboration, how it works and how their participation is essential to its success.

### Strengthen publicity on collaborative services

As things stand, publicity about hospital–community health centre collaboration is inadequate, resulting in low awareness among patients and even among health professionals. The government and relevant health organisations need to strengthen publicity on hospital–community health centre collaborations and the services they provide. Different publicity strategies may be needed for various target audiences, including the general public, patients with different educational levels, physicians and other care providers.

### Sharing of experience

Since hospital–community health centre collaborations in China started more than a decade ago and since different models of collaboration have emerged, it is time for governments, healthcare institutions, collaborations and researchers to share experience, compare notes with respect to the merits of different models and discuss possible future developments. Instead of working in isolation, hospital–community health centre collaborations need to collaborate and learn from each another in order to move forward. It is hoped that our exploratory study provides an impetus to start the dialogue among hospital–community health centre collaborations. As well, China should seek to exchange information with other nations as it could benefit from the experience of other countries where similar collaborations are in place.

## Competing Interests

The authors declare that they have no competing interests.
